# Book Reviews

**Published:** 1801-09

**Authors:** 


### CRITICAL RETROSPECT OF MEDICAL AND PHYSICAL LITERATURE.

---

*Genius der Gesundheit und des Lebens;* i. e. Genius of Health and Life, a Pocket Book for Phyficians and others, for the Year 1801, by Dr. C. J. Kilian. Leipzig, Wiegel, price 16 grofhen, or about 2s. 6d.

This publication is intended for the ufe of the public at large, in order to divulge better and more enlightened notions on objects of medicine, by which means the author, who occupies an honourable place amongft the medical writers of Germany, thinks to banifh feveral prejudices that are ftill reigning amongft the people, with refpect to medical affiftance. For this purpofe he here gives: 1. *Newest theory of general medicine, theoretical as well as practical.* We doubt very much whether this Memoir will anfwer the purpofe, as it is too philofophically written to be fufficiently underftood by every reader; and it feems to us not at all proper to propofe an entire fyftem of medicine in a popular work, as by being frequently mifunderftood, it is apt to convey wrong and imperfect ideas to the reader. 2. *Fragments of a domestic materia medica.* It is the defign of the author to give a furvey of all the domeftic remedies, which will be yearly continued. This is indeed a laudable undertaking, as that part of medicine has been hitherto very little attended to. The author treats here of milk and eggs. 3. *Some dietetic observations and precautions for smoakers.* No perfon will wonder at finding fuch an article, who knows that the cuftom of fmoaking tobacco is ftill prevalent in Germany amongft the higher claffes of people; rules, therefore, concerning that habit, are not foreign to this publication, and they will be found entertaining, as the author gives them with much wit and humour. 4. *Two medical observations.* 1. On the effects of bathing in rivers, which, in fome cafes, is very prejudicious to health. 2. On the ufe of bifhop, (a liquor compofed of red wine, fpices, fugar, &c. like negus) in a cafe of hæmoptoë.

---

*A Treatise on the Cow-pox, fully explaining its Causes, Symptoms, Inoculation, Treatment relative to other Eruptions on the Skin of Men and Beasts, according to Observations made by the Author.* By Frederick Benjamin Osiander, M. D. Profeffor of Medicine and Midwifery in Gottingen. With coloured copper-plates. Gottingen, 1801, octavo, pp. 238.

The author is the firft phyfician who inoculated with the Cow-pox fuccefsfully in Gottingen, and earneftly recommended it by powerful arguments in an exprefs publication. The copper plates accompanying it, which are well engraved and carefully coloured, rerepresent represent the hand of a milkmaid having a Cow-pock, and also another plate in folio, representing the course of the daily appearance of the Cow-pox, according to Prof. Osiander's method of inoculating with a blister.

The drawing is done from Nature by Dr. Osiander himself, and the engraving by Basemann, a good painter and engraver in Gottingen. It is a very exact delineation of the Cow-pox pustule on the place of inoculation, in its various changes throughout the whole course of the disorder.

---

*An Essay on the Proximate Cause of Animal Impregnation, being the Substance of a Paper read and discussed in the Medical Society at Guy's Hospital, in October,* 1799. By JOHN PULLEY, of Bedford, Member of the Royal College of Surgeons, London. 4to. pp. 31. London, 1804, Cox, &c.

Mr. P. in his Preface says, "It is the sole intention of the author to prove by arguments and facts, the inefficacy of the doctrines taught by Dr. Darwin and Dr. Haighton, on the mysterious subject of animal impregnation;" and not those two physiologists only, but "all who give exclusively to one sex the power of reproduction."

"In tracing the process of generation," says Mr. P. "theorists have ranged themselves on three distinct grounds, each of which has had its ardent advocates, and as strenuous opponents. One gives to woman alone the humble office of affording a proper nidus for the due evolution of the fœtus, which according to this theory already exists in the male semen, and requires only a fruitful habitation. Another directly reverses this position; it puts the female in possession of every requisite for the formation of a new animal, and considers the male a mere stimulating engine to call the latent powers of the female into life. The third gives not pre-eminence to either sex, but with the mutual embrace produces a mutual effect; it regards both the male and female as most essentially concurring in the work of reproduction, each affording a something, which, uniting under proper circumstances, becomes the proximate cause of impregnation.

"Although every existing theory on the reproduction of animals is reducible in its principle to one of the above grounds, the warm and fertile imagination of speculative minds has led to almost innumerable modifications, each theorist assuming his fundamental position, and forming his deductions in manner and respect to the direction of his fancy."

After combating the opinion that *corpora lutea* are decisive proofs of impregnation, he says, "What a train of evidence, embracing facts most positive and indisputable, does that author call up against him, who maintains that the male semen alone possesses the power of stimulating the os uteri and adjoining parts, and that by sympathy generation is effected. When a negro man embraces a white woman, why is it that the offspring is a mulatto? When a male ass copulates with a mare, why does the mule partake of the nature nature of both? And when dogs and bitches of different species have intercourfe, why in appearance do the mongrel whelps claim affinity to both parents? Again, it is a felf-evident truth that a child may inherit the difpofition to the conftitutional difeafes of either parent; and fhall it be faid, that it is in the power *of sympathy* to hand down to pofterity the contaminated habit of the father? Befides, if the femen be allotted merely to ftimulate the uterine fyftem, it would feem a totally unneceffary fecretion; for we find that the fexual act is not wanting, even to effect thofe changes, which the femen, by this theory, is only permitted to perform."

---

*A Treatise on Febrile Diseases, including Intermitting, Remitting, and Continued Fevers; Eruptive Fevers, Inflammations, Hæmorrhagies, and the Profluvia; in which an Attempt is made to present, at one View, whatever, in the present Sate of Medicine, it is requisite for the Physician to know respecting the Symptoms, Causes, and Cure of those Diseases.* By A. P. Wilson, M. D. F. R. S. Ed. Fellow of the Royal College of Phyficians, Edinburgh, &c. Vol. II. 8vo. pp. 580. Winchefter, 1800. London, Cadell and Davies, Callow, &c.

Our readers will fee our account of the Firft Volume in the fecond vol. of the Med. Journal, p. 300. That volume having exhaufted intermittents and continued fevers, Dr. W. poftpones the Phlegmafiæ of Dr. Cullen, and in this fecond volume completes his First Part, or *Idiopathic Fevers*, with the *Exanthemata*.

In the preface to this fecond volume, Dr. W. replies to fome criticifms on the firft, and, as we predicted, his obfervations on the Brunonian fyftem and nofology have not efcaped notice.

He replies, "The Brunonian doctrines have been fo warmly contefted, and fo frequently miftated, that I examined them with a degree of caution and minutenefs, which would not otherwife have been neceffary. And aware as I was of the hypothetical manner in which the proximate caufe of fever had been treated by every other writer as well as Dr. Brown, it will be admitted, I hope, that I have not departed from a due degree of caution in any of my obfervations on this fubject.

"With all the care I was capable of, however, I have not fucceeded in conveying the fame ideas to every reader. It has been ftated by one, that I efpoufe the general principles of the Brunonian fyftem; by another, that I admit no part of it but that which was admitted by all phyficians, before Dr. Brown's Elements of Medicine appeared. By one, many of my objections to this fyftem are regarded as invalid; by another, their validity is admitted, and I am cenfured for allowing it any merit at all. I am faid by one to aim at extending the Brunonian fyftem; by another, accufed of attempting to bring about a coalition between the fyftems of Dr. Cullen and Dr. Brown, which the critic more juftly than elegantly obferves, is as hopelefs a tafk as endeavouring ing to milk he-goats. What ſhall I ſay in anſwer to ſuch contradictory objections? I ſhall only obſerve of the two laſt, that I am perfectly unconſcious of having made either the one attempt or the other. All I have attempted is to give an accurate view of the Brunonian ſyſtem, to ſeparate the true from the falſe parts of it, and to arrange certain facts relating to the laws of excitability without reference to any ſyſtem whatever. I ſhall here endeavour in a few words to place the reſult of what was ſaid of the proximate cauſe of fever in a clearer point of view.

"When a ſtate of exceſſive excitement or atony exiſts independently of the continued application of ſome artificial agent, one of two changes muſt have taken place; either the quantity or quality of the natural agents, or the ſtate of the living ſolid, is different from that which prevails in health. If it can be ſhown that the ſtate of the living ſolid remains the ſame, it follows that the deviation from health is owing to ſome change in the natural agents; if it can be proved that the ſtate of theſe agents remains the ſame, it then follows, that the deviation from health is owing to ſome change in the ſtate of the living ſolid. We may go a ſtep farther; if it can be proved that ſome of the natural agents remain unchanged, and yet produce effects different from thoſe they produce in health, it not only follows that the ſtate of the living ſolid is changed, but alſo that, if this change in the ſtate of the living ſolid will account for the changes obſerved in the effects of other natural agents, we are not in any degree to attribute ſuch effects to a ſuppoſed change in theſe agents, there being no occaſion for any ſuch hypotheſis to explain the phenomena. In fever, many of the natural agents, caloric, food, light, noiſe, for example, evidently remain unchanged, the difference in their effects, therefore, is owing to a change in the ſtate of the living ſolid. But this change is capable of accounting for the change we obſerve in the effects of thoſe agents whoſe condition we cannot with preciſion aſcertain, the circulating and other fluids. It follows, therefore, that whatever change may take place in theſe during the progreſs of fever, and however this change may modify the ſymptoms of fever, too great lentor, acrimony, or other morbid condition of the fluids, is not the proximate cauſe of fever.

"With reſpect to the hypotheſis of fever depending on a change in the ſtate of the ſimple ſolid. As the natural agents act not on the ſimple but on the living ſolid, it is neceſſary to ſuppoſe a change in the ſtate of the latter; and as this change accounts for the phenomena of fever, there is no occaſion for any other ſuppoſition.

"And farther, as all the natural agents excite a morbid action, and as this effect is not confined to any one, but obſerved equally in every part of the ſyſtem, what room is there for ſuppoſing that any one part is more particularly affected than every other?

"Laſtly, with regard to fever being a ſtate of accumulated or exhauſted excitability, in the ſenſe in which Dr. Brown uſes theſe terms, terms, it is only neceſſary to refer to the facts, which prove that no ſuch morbid ſtates exiſt. It is true that the phenomena of ſynocha are ſuch as we ſhould expect from an accumulation of excitability; but will a ſurfeit, or an exceſſive quantity of diſtilled ſpirits, frequent cauſes of ſynocha, occaſion an accumulation of excitability? It appears then, that in fever the living ſolid is ſo changed, that a change is effected in the laws of its excitability, and that this admitted, there is no occaſion for any of the foregoing hypotheſes to explain the phenomena eſſential to fever. Upon the whole then, the following, as far as it goes, would appear to be a juſt view of the nature of fever.

"Every agent acting on the ſyſtem in general is capable of producing three effects, moderate excitement, exceſſive excitement, or atony, according to the degree in which it is applied. The firſt operation of agents conſtitutes health; the two laſt general diſeaſe, which has been called fever. If, by the application of artificial, or the exceſſive application of natural agents, either of the two laſt ſtates be maintained for a ſufficient length of time, the living ſolid is ſo changed, that is, ſuch a habit is formed, that the natural agents applied in the uſual degree produce the ſame morbid effects till the diſeaſed habit has been counteracted; which, as in the caſe of other habits, is the more eaſily effected, the ſhorter its duration has been. Hence it is that almoſt any thing making a ſtrong impreſſion will ſometimes remove fever at an early period; and hence the difficulty of removing a fever is generally proportioned to the time it has laſted. The means which cure a fever at an early period, that is, produce a criſis, ſeem either to expel the offending cauſe before the morbid habit is effected, as vomiting during a fit of drunkenneſs; or break the morbid habit before it has gained force, as cold-bathing during the firſt days of fever. In a more advanced ſtage, as the morbid habit is corrected with more difficulty, it is corrected more ſlowly. When in ſynocha we ſucceed in changing exceſſive into moderate excitement, h. e. into that excitement which is followed by exhauſtion, we have removed the morbid habit, and conſequently cured the fever. The cure of ſynocha, therefore, depends on the abſtraction of ſtimuli. But as atony is the conſequence of exceſſive excitement, if exceſſive excitement has laſted for a conſiderable length of time, atony will always be evident, previous to the reſtoration of health. Hence it is, that the ſymptoms of typhus ſucceed thoſe of ſynocha. When we ſucceed in changing atony into moderate excitement, we have corrected the morbid habit, and conſequently cured the fever. The cure of typhus, therefore, depends on the addition of ſtimuli.

"I have been indirectly accuſed of having aſcribed too much importance to the ſtudy of noſology. It is faſhionable at preſent to regard noſology as a very uſeleſs branch of medicine. Will the knowledge of a noſological ſyſtem, it is ſaid, enable you to cure a diſeaſe? It certainly will not, but it greatly aſſiſts in acquiring the knowledge that will. Let thoſe who ſlight the labours of the nofologift, recollect that it is his province to point out the fymptoms which diftinguifh one difeafe from another, and to arrange difeafes in fuch a way as may beft fhew their affinity, and confequently affift the memory in recollecting their modes of treatment. The anatomift detects the changes induced by internal difeafe; but of what ufe would this knowledge prove, did not the nofologift point out the means of afcertaining the prefence of fuch morbid ftates previous to death. In vain might the chemift and botanift fupply us with medicines, did not the nofologift enable us to diftinguifh the cafes in which they are ufeful.

"It has been afferted, that the practice of medicine would be improved by attending to fymptoms individually, without attempting to afcertain their various combinations, and applying to thefe combinations particular names. Thofe who make this affertion maintain, and if the affertion is true, juftly maintain, that nofology is an ufelefs ftudy. Is the affertion true? Does any fymptom at all times require the fame mode of treatment? Nay, is not the fame fymptom in one cafe falutary, in another pernicious? We muft, therefore, be influenced in treating each fymptom by an attention to thofe which accompany it, that is, an attention to the combinations of fymptoms is neceffary, and confequently nofology of the firft importance. By far the greater number of miftakes I have witneffed in practice, have originated from the neglect of nofology. It often happens that an opinion, at firft maintained on no other account than its fingularity, becomes current among thofe who are unable, or will not be at the trouble, to think for themfelves. Many exclaim againft nofology, but cannot tell why. The truth is, an accurate knowledge of it is acquired with difficulty, and the indolent are glad of an apology for neglecting it altogether."

He concludes his preface thus:

"Contrary to my firft intention, the prefent volume includes all the fpecies of eruptive fevers, and confequently finifhes the firft part of the work, comprehending idiopathic fevers. The two volumes now publifhed, therefore, form a Treatife on Idiopathic Fevers, and may be regarded as independent of thofe which are to follow.

"The Symptomatic Fevers will form the three remaining volumes. In the firft of which, that is, the third volume of the work, the inflammations of the fkin, head, and neck, will be confidered; in the fourth, thofe of the thoracic and abdominal vifcera, and of the joints. The laft volume will comprehend the hæmorrhagies and profluvia, with a more detailed view of the nofology of febrile difeafes.

"It is my intention, when the prefent work is finifhed, to commence another, (which will confift of two volumes, and for which the materials are already collected) on the Nervous Complaints moft frequently complicated with Febrile Difeafes."

In the volume before us, the author, in concluding his fecond book, treats of the *varieties* of continued fever, viz. petechial, miliary, aphthous, veficular, and eryfipelatous.

The third book contains the Exanthemata, viz. variola, vacciola, varicella, rubeola, fcarlatina, peftis, and urticaria.

As our readers have probably more curiofity refpecting the Plague than either of the other difeafes treated of in this volume, we fhall prefent them with a few extracts on that fubject:

"*Of the Plague.*

"Although few Britifh phyficians have occafion to practice in the plague, the propriety of being acquainted with a difeafe, which has demanded fo much attention, and bears fo ftrong an analogy to complaints which every day fall under their care, is too apparent to require any comment. Befides, we cannot forefee in what circumftances we may be placed; and for a phyfician to betray ignorance of the plague would be unpardonable.

"There are few difeafes fo remarkably varied, and there are few, perhaps none, of which it is more difficult to give an account, which fhall be at the fame time fufficiently full and diftinct. The reader will find the beft writers, who have had an opportunity of feeing the difeafe, complaining of this difficulty.

"As much as poffible to prevent confufion, they have divided the plague into different claffes; nor is it poffible without this to give a juft view of the complaint, fince no two difeafes are more oppofite than the different forms of the plague. In one we fhall find it the moft dreadful of all fevers, deftroying without exception all whom it attacks; in another we fhall find it confifting chiefly of an eruption unattended by danger. Why, it may be faid, are difeafes fo different, regarded only as varieties of the fame? However different thefe extremes, they are not only produced by the fame fpecific contagion, but almoft infenfibly run into each other; from which we may form fome idea of the variety which the plague prefents. It will not then appear furprifing that the difficulty of arranging its fymptoms, fo as on the one hand to avoid confufion, and on the other to give a comprehenfive view of the complaint, be very great. In fact, there is no author, although moft of thofe who have written on the plague fpeak from their own obfervation, who has overcome this difficulty. In all, we find the account either fhort, and confequently more or lefs imperfect, or of confiderable length, and more or lefs confufed. So varied are the fyptoms of the plague, that if the common varieties are given, the account muft be tedious, and then it is impoffible perhaps to prevent it being in fome degree perplexed.

"I fhall not fpend time by laying before the reader the modes of divifion adopted by different writers, or by pointing out the objections which might be made to them. The objections confift chiefly, in many of the divifions not being marked with fufficient precifion, fo that it is often impoffible to fay what are the correfponding divifions in the different accounts of the difeafe; as the reader will perceive if, for example, he compare together the different accounts of the plague in the Traité de la Pefte, or any of

 thefe thefe with the divifion adopted by Dr. Ruffell, in his Treatife on the difeafe.

" In dividing a difeafe into varieties, each variety muft be marked by fome fymptom which conftantly attends it. It muft not be accidental or unconnected with the ftate of the fymptoms in general, which would render the divifion ufelefs; but muft mark a variety, in which the fymptoms on the whole, and, what renders the divifion of more importance, the prognofis, differ from thofe of other forms of the complaint.

" I fhall not defer a particular account of the eruptions, namely, the buboes, carbuncles, &c. till after the different forms of the difeafe have been confidered, as has ufually been done; by which we are forced to ufe terms before they have been defined; nor on the other hand, is it proper, where the variety is fo great, to interrupt the account of the general courfe of the difeafe, in order to defcribe the different eruptions. It therefore appears neceffary to depart from the order which has been purfued in laying down the fymptoms of the other exanthemata, and to regard bubo, carbuncle, &c. as terms which muft be defined, before proceeding to give the fymptoms of the plague.

" *Of Peftilential Eruptions.*

" 1. Of PESTILENTIAL BUBOES.

" A peftilential bubo at its commencement is a fmall, hard, round tumour, readily perceptible to the touch, about the fize and fhape of a pea, it is moveable under the fkin, the appearance of which is not altered at an early period, the bubo lying at a greater or lefs depth, and the fwelling not appearing externally.

" As the tumefied gland enlarges, it changes from a round to an oval fhape, becoming at the fame time lefs moveable. The integuments now begin to thicken, and the fwelling to appear externally.

" The appearance of the bubo is often preceded by a fenfe of tightnefs and pain fometimes lancinating, or itchinefs, in the part where it is about to appear, now and then by fhivering. In many cafes, however, the fmall fwelling juft defcribed comes on without being preceded by any peculiar fymptoms.

" Some buboes are indolent and infenfible, others very fenfible and rapid in their progrefs. The tumour advancing quickly to fuppuration, is generally regarded as favorable. When the buboes fuppurate properly, De Mertens obferves, and there is a feparation of efchars from the carbuncles, with a remiffion of the febrile fymptoms, the prognofis is good. No general rule however can be laid down. Cafes where early fuppuration takes place often prove fatal; and there are many hiftories of cafes terminating favorably where the buboes were extremely indolent and terminated in refolution.

" It is difficult to forefee in what way a bubo will terminate. The fluctuation is often fcarcely perceptible where fuppuration has taken place, and buboes are fometimes refolved after fluctuation has has been very evident. Their progrefs indeed is almoft always more or lefs irregular, efpecially after the firft week. At one time they feem advancing to fuppuration, at another fhow a tendency to refolution. 'But thefe variations,' Dr. Ruffell remarks, 'chiefly 'refpected the integuments; for the gland itfelf when carefully 'explored was feldom found to alter, and where the tumour ac- 'tually difperfed, it was not fuddenly, but by flow degrees. Thus 'from the alteration in the teguments alone, the whole tumour, on 'a fuperficial view, feemed to leffen or increafe, though the gland 'remained the fame; and I am inclined to think that this deception 'was often the caufe of the bubo being faid to fluctuate, or to 'vanifh in appearance entirely, and again return.' He adds, however, 'At the fame time I am far from thinking that this fluctua- 'tion was never real.' And Chenot obferves, 'Vidimus quoque 'abruptam fuppurationem in his refufcitari ac demum per effufio- 'nem puris abfolvi.'

"The bubo as it increafes in fize becomes fomewhat flat; and generally about the fecond week the fkin over it grows tenfe and painful, and begins to be inflamed. In fome cafes the inflammation is moderate, in others confiderable; but it feldom terminates in gangrene, although the fkin now and then affumes a bluifh colour.

"It fometimes happens, however, that the bubo runs to fuppuration without any degree of inflammation appearing on the fkin, and then, as it is generally harder than a fuppurated venereal bubo, it is often difficult to determine whether fuppuration has taken place or not. When buboes break fpontaneoufly it generally happens in the third week, fometimes at a later period.

"The buboes moft frequently appear in the groins or a little lower, among the loweft clufter of the inguinal glands; they alfo frequently appear among the axillary glands; fometimes, though more rarely, they have their feat in the parotid, and the difeafe is then by many reckoned more dangerous than when the buboes appear in the groins or arm-pits. Still more rarely they appear in the maxillary or cervical glands.

'The latter too,' namely, the maxillary and cervical glands, Dr. Ruffell remarks, 'were feldom obferved to fwell without either the 'parotid fwelling at the fame time or foon after, or a carbuncle pro- 'truding near them; they never were the fole peftilential eruptions, 'and I recollect few inftances of their coming to maturation.' It has been remarked by others, that the parotid bubo feldom appears unaccompanied by one or more in the axilla or groin.

"It may upon the whole be obferved, that the axillary buboes fuppurate more frequently than thofe fituated about the fauces, and the inguinal more frequently than the axillary.

"Buboes often make their appearance on the firft day of the complaint; fometimes indeed they are among the firft fymptoms. It has been obferved, that when they appear later than the third or fourth day, they are generally preceded by an exacerbation of the febrile fymptoms. Thofe which come out at fo late a period, however, however, are not, for the moſt part, the firſt which appear in the courſe of the complaint; for a ſucceſſion of buboes ſometimes takes place, till three or four have made their appearance. In this caſe ſeveral hours uſually intervene between the appearance of any two of them.

" It ſometimes happens that no buboes appear, and theſe caſes are upon the whole the moſt fatal. This is a circumſtance which particularly demands attention, as the caſes unattended by buboes and other peſtilential eruptions generally make their appearance at the commencement of the epidemic, and have often, in conſequence of the abſence of the eruptions, been miſtaken for other complaints. In other caſes, particularly towards the decline of the epidemic, the buboes and other eruptions often form the principal part of the complaint, which is then unattended by danger; from which it would appear, that the eruptions in the plague are to be regarded as favorable ſymptoms; but of this I ſhall preſently have occaſion to ſpeak more particularly.

" Where the inflamed gland advances to ſuppuration more rapidly than the integuments, troubleſome fiſtulous ulcers are ſometimes formed, if an artificial opening has not been made in the ſkin. This accident however is rare; in general the buboes, left to themſelves, do not prove troubleſome.

" When they do not ſuppurate, and the patient recovers, they gradually diſperſe, generally in the ſpace of a few weeks. In ſome caſes they are ſucceeded by an induration of the gland, which remains for many months. Even where ſuppuration has taken place, if the cure proves tedious, either in conſequence of the matter having been diſcharged by too ſmall an opening, or the opening having repeatedly cloſed in the progreſs of the cure, a ſimilar induration ſometimes ſucceeds, which in like manner ſooner or later diſappears, theſe indurations never terminating in cancer.

" Such are the circumſtances to be learned from attending to the external appearances of the buboes; ſome further circumſtances, of leſs moment however, have been aſcertained by diſſection.

" It has been the practice of many, particularly the French ſurgeons, to extirpate the buboes, which gave them an opportunity of obſerving the internal changes which take place in them.— From the appearances on diſſection they have been divided into ſeveral different ſpecies. It is unneceſſary to detain the reader with an account of this hitherto uſeleſs diviſion; he will find it at length in the Traité de la Peſte from the 428th to the 434th page. One obſervation deſerves attention; it has juſt been remarked, that the ſkin covering the buboes never runs to gangrene; diſſection ſhows that it is otherwiſe with reſpect to the gland itſelf, ' Je coupai par le milieu celle (h. e. the bubo) qui etoit ſur les ' vaiſſeaux, que je trouvai toute noire. Le lendemain j'ouvris le ' bubon, j'y trouvai le corps glanduleux comme un rein de mouton, ' tout noir.'

" Beſides the true bubo, another peſtilential eruption has alſo received the name of bubo. This eruption is ſo rare that ſome who who mention it have been accufed of mifreprefentation. This accufation we are now affured is groundlefs.

"The principal circumftance in which the fpurious differs from the true bubo, is in the former appearing indifcriminately on almoft every part of the body, while the true bubo is confined to the groin, axilla, and parts about the fauces. 'Spurious buboes 'were obferved,' fays Dr. Ruffell, 'on the head, the forehead, 'the throat, the fhoulder, above the clavicle, the neck, on or 'above the fcapulæ, the back, the fide under the breaft, the belly, 'the hip, hind part of the thigh near the ham, the leg, the 'fcrotum, the arm near the ufual place of iffues, infide of the arm 'near the elbow, outfide of the fore arm, and near the wrift.'

"Some of thefe buboes, if they are not lanced at a proper time, grow to a great fize, particularly thofe on the fcapulæ or back; in other parts, however, they feldom much exceed the fize of a common hen's egg. They generally make their appearance about the fecond or third day, and for the moft part after the protrufion of true buboes or carbuncles. They generally fuppurate, though lefs rapidly than the true buboes.

"2. Of CARBUNCLES.

"Next to buboes, carbuncles are the moft remarkable of the peftilential eruptions.

"The reader will find carbuncles divided by different writers into feveral varieties. One makes three, another four, a third five different kinds.

"Dr. Ruffell divides the carbuncles he met with, into five varieties.

"The firft appeared in the form of a fmall puftule about the fize of half a pea, on its upper furface of a dufky or yellow colour, and a little wrinkled. The fkin which immediately furrounded this puftule was hard and inflamed. The puftule itfelf foon became very painful, and continued to increafe till it became a tumour of the fize of a nutmeg, and fometimes that of a walnut, and a yellowifh matter was fecreted under the cuticle, which was fometimes moift, at other times dry and crufty; the reft of the tumour affumed a dark reddifh colour, the circle which furrounded it appearing at different times of various hues.

"On the third, fourth, or fifth day of the carbuncle, a gangrenous cruft appeared on the middle of it, which foon occupied the whole furface of the tumour, exactly refembling the black efchar formed by cauftic.

"This cruft, when the termination was favorable, was thrown off by fuppuration, leaving an ulcer of various depth, which for fome time continued to difcharge matter. When the cafe terminated fatally, the cruft remained dry and often fpread to the inflamed circle, furrounding the carbuncle, fo as to form a gangrene of confiderable extent.

"The fecond kind of carbuncle appeared in the form of a fmall angry puftule, not rifing fo high as the former, more difpofed to fpread, and becoming gangrenous on the fecond day. In this ftate it was not eafily diftinguifhed from the other, but was generally furrounded with a more highly inflamed ring. It chiefly attacked tendinous parts, particularly the joints of the fingers and toes.

" In the third variety, the cuticle was at once raifed into a blifter of the fize of a horfe bean, filled with a dufky yellow or blackifh fluid, and the fkin which furrounded this variety of the carbuncle was lefs tenfe and of a paler red than that furrounding either of the foregoing. When the blifter broke, the cuticle fell upon the flat furface, which was of a dark colour and foon became black. At this period, that is, about the third or fourth day of the carbuncle, it refembled the preceding varieties, except that it was flatter. The circle furrounding the efchar gradually affumed a very dark red, but never became gangrenous. The efchar was about the fize of a fix-pence. This carbuncle was very painful, and five or fix fometimes appeared on the fame patient.

" The fourth variety was a fmall red fpot raifed only to the touch, which gradually rofe higher and fpread, till in twenty-four hours it was a flattifh dufky puftule, furrounded by a light rofe-coloured margin. This carbuncle was very painful, and when it appeared on the face occafioned fwelling, but without inflammation of the fkin. It often became black beyond the rofe-coloured margin on the fecond day, and the mortification fpread to the neighbouring parts. This fpecies of carbuncle always accompanied other eruptions, and was ufually pretty numerous.

" The fifth and laft variety appeared at firft a puftule, which, on the fecond day, refembled that of the fmall-pox; it rofe in the form of a cone to twice the fize of a large diftinct pock with a blunt yellowifh point, which, inftead of advancing to fuppuration, became black to the fize of a large field pea. The gangrene in this cafe however did not fpread farther. The margin became of a dufky red, but appeared brighter as the fuppuration, which threw off the efchar advanced. After the fecond day, this differed from the third and fourth varieties only in the gangrenous part being of lefs extent, and the puftule more raifed.

" There are certain eruptions which now and then appear in the plague in fome refpects differing confiderably from any of the carbuncles juft defcribed; in others refembling them. Such is the eruption which has been termed papulæ ardentes, or fire bladders.

" But it would be tedious to enumerate all the various eruptions of this kind which have been obferved in different epidemics.— The true peftilential carbuncle may be defined, a puftular or veficular eruption, fooner or later running to gangrene.

" The eruption called anthrax, is nothing more than a carbuncle after it has become fphacelated.

" Carbuncles, to whatever variety they belong, for the moft part do not exceed the fize of a walnut; they have fometimes been obferved confiderably larger. The time of appearance is uncertain; they fometimes fhew themfelves on the firft day of the complaint, but more commonly not till a later period; and when feveral appear pear on the fame perfon, they generally fucceed each other rapidly. They have been known to come out as late as the eighteenth or twentieth day.

"Refpecting the number which appears on the fame patient, Dr. Ruffell obferves, 'Of thofe of the firft and fecond fpecies fel-'dom more than one or two were obferved in the fame fubject, in 'general one only. The other varieties occurred in greater num-'ber, and including thofe of the fifth, I have fometimes counted 'between twenty and thirty, but this happens very rarely.'

"This eruption is always attended with confiderable pain, which in fome cafes is very violent. No external part of the body is exempted from carbuncles. 'I have obferved them every where,' the author juft quoted remarks, 'the penis and fcrotum not ex-'cepted, but never obferved them on the tongue, the tonfils, and 'internal parts of the mouth,' (there have been inftances however of their appearing on the tongue) though in carbuncles on 'the 'cheek, near the corner of the mouth, the gangrene fpreads in-'wards, and in one inftance of a carbuncle on the eye-brow, the 'gangrene fpreading upon the globe of the eye had deftroyed part 'of it.'

"The carbuncle is a lefs favorable eruption than the bubo. Carbuncles were regarded by the Ruffian phyficians, Dr. Guthrie informs us, as a fign of greater malignity than buboes; but of this prefently. They thought the carbuncle indicated lefs danger when red than when livid; when it fuppurated than when it did not. When the hands and feet were the feat of carbuncles, Dr. Guthrie obferves, the patient feldom or never recovered. Carbuncles on the fpine were alfo regarded as particularly unfavorable."

"*Of the other Symptoms of the Plague.*

"That I may abridge the following account of the fymptoms of the plague, and confequently render it more diftinct, it is proper to obferve, that all the fymptoms both of fynocha and typhus occafionally attend this fever. It is unneceffary again to detail all of thefe, for which I refer the reader to the firft volume; and fhall here confider at length, thofe which characterife the plague, or appear in this difeafe under peculiar modifications.

"It has already been remarked, that in the feveral divifions of the plague adopted by writers, many of the varieties are ill defined. They feem to be marked by accidental fymptoms, and fome of them by no particular fymptom, but by the general mildnefs or feverity of the difeafe. In the former cafe the divifion can be of no ufe; in the latter it can admit of no precifion. Befides, moft writers on the plague, fpeaking from their own obfervation alone, defcribe the complaint as it appeared in one or two epidemics, and in almoft all epidemics there are peculiarities. It is only by comparing many, that we can form an account of the difeafe generally applicable.

"On comparing different epidemics, we fhall find, that whatever be true of other eruptions, buboes are falutary. They almoft always mark a form of the diſeaſe leſs generally fatal than that unattended by this eruption. ‘ Thoſe periſhed,’ Dr. Ruſſell remarks, ‘ ſometimes within the twenty-four hours, ſometimes on ‘ the ſecond or third day; they had neither buboes nor carbuncles, ‘ and it was very rare to find ſuſpicious marks of infection on the ‘ dead bodies.’ In another place he obſerves, ‘ the total abſence of buboes in thoſe who died ſuddenly I have doubt of.’ He alſo remarks, ‘ that the plague, under a form of all others the moſt ‘ deſtructive, exiſts without its characteriſtic eruptions or other external marks reckoned peſtilential, can admit of no doubt.’ The Ruſſian phyſicians, Dr. Guthrie informs us, found the caſes attended with buboes leſs fatal than thoſe attended with carbuncles. Carbuncles and petechiæ, De Mertens, in his account of the Plague of Moſcow, obſerves, are not critical eruptions, they only denote a putrid condition of the humours; whence it follows, that in proportion as buboes are more common, and petechiæ and carbuncles more rare, the milder is the plague. Orrœus, in his Treatiſe on the ſame Plague, obſerves indeed, that buboes often attended the moſt acute form of the diſeaſe; yet in another place he informs us, that there were no buboes in the worſt form, their germs only being ſometimes obſerved after death; and Samoilowitz, in his account of this epidemic, in deſcribing the worſt form of the diſeaſe, notices petechiæ and carbuncles as frequent ſymptoms, but makes no mention of buboes.

“ Upon the whole, then, the plague unattended by buboes, runs its courſe more rapidly, and is more generally fatal, than when accompanied by this eruption. The plague may therefore be divided into that which is, and that which is not, attended with buboes.

“ The firſt of theſe includes many varieties, from that in which the prognoſis is almoſt uniformly good to a form of the diſeaſe little leſs fatal than that unattended by buboes. The appearance of the buboes alſo affords the means of ſubdiviſion, for it will be found, on comparing the accounts of different epidemics, that upon the whole the earlier the buboes appear, the milder is the diſeaſe; thus, for example, in the firſt claſs of Dr. Ruſſell's diviſion, which was the moſt fatal, buboes were very rare; in the ſecond, which was alſo very fatal, though leſs uniformly ſo, buboes appeared on the third day or later; in the third claſs, which was leſs fatal, they appeared earlier; in the fourth, which was ſtill milder, buboes generally appeared on the firſt day; in the fifth, which never proved fatal, they were among the firſt ſymptoms of the complaint.

“ To the two foregoing forms of the diſeaſe a third might be added, ſince the peſtilential eruptions towards the end of the epidemic ſometimes appear unattended by fever. This form, however, which is merely a local affection unattended by danger, demands little attention.

“ The moſt fatal form of the plague makes it attack in various ways, ſometimes merely with depreſſion of ſtrength, a ſenſe of weight weight in the head, confusion of thought, giddiness, dejection, and oppression about the præcordia, often accompanied with a bitter taste in the mouth. * The patient is inclined to be silent, shews much anxiety in his countenance, but makes few complaints; the febrile symptoms are very moderate. The attendants suppose the patient a little indisposed, but suspect nothing alarming; yet such patients often die within the first twenty-four hours, sometimes on the second day.

"In general, however, this form of the plague makes its attack less deceitfully. In an epidemic described by Chenot, that of Marseilles, and many others, the symptoms from the first were alarming, the complaint often appearing with violent and irregular shaking.

"Delirium is sometimes the first symptom observed. At other times, a remarkable state of the pulse, which very suddenly becomes so weak that it can hardly be felt, frequent and intermitting, with much debility and languor, introduces the disease. The prostration of strength is sometimes so sudden and complete, that Mr. Smith, Dr. Guthrie informs us, saw men in apparent good health, on being infected by the plague, suddenly drop down as if shot by a musket ball. Sometimes, instead of mere debility and lassitude, the patient is affected with extreme horror and despair, and his spirits sink so low that nothing can recall them.

"At other times, the disease attacks with very slight chills, soon followed by a burning heat, which remains during the disease; as soon as the heat commences, the patient complains of insufferable head-ach and excessive thirst. Sometimes, as in the plague of Russia, described by Orrœus, the patient is suddenly seized with violent shivering succeeded by a hot fit, the shivering and hot fit alternating several times.

"The first symptom of the plague is sometimes a violent beating of the temporal arteries, while the pulse at the wrist is small and feeble. In this case the heat is generally moderate, but the head-ach intolerable. In the plague which raged at Lyons in 1628, a burning heat in some of the viscera, and a dull pain, or rather great heaviness of the head, announced its approach.

"The plague sometimes comes on with violent palpitation, and strong convulsive tremblings. The plague which raged in London in 1665, often made its attack in this way.

"As the complaint advances, it assumes more of the appearance of the fevers we have been considering. The inflammatory symptoms generally run high for the first day or two; but for the most part, the plague assumes the form of typhus at

---

* The bitter taste in the mouth the reader will find mentioned by different writers as characteristic of the plague. It is observed by some, that a favourable change seldom happened while this symptom continued.

an early period; and the patient soon becomes delirious or comatose.

"The delirium is sometimes of the furious kind, particularly, Orrœus observes, in those of a robust and full habit, and in whom a full meal appeared to be the immediate exciting cause. In general, however, the delirium is of that species which characterises typhus, the patient appearing rather stupid than outrageous, and complaining of a pain at the heart, a symptom frequently observed in the plague. When coma comes on early, it has been looked upon as affording a worse prognosis than delirium, particularly if it suffers no evident remissions during the day time. Both delirium and coma indeed are almost always most considerable during the night. The remission in the day time is generally more evident when the patient is delirious than when he is comatose.

"Whether he becomes comatose or not, there is always present a very remarkable muddy appearance of the eyes, which is sometimes observable at the very commencement, and is one of the most characteristic symptoms of the disease. This appearance of the eyes in some degree resembles that in the last stage of malignant fevers. It is not, however, described as altogether such, for with the muddiness there is blended a degree of lustre. It is an appearance in short very remarkable to those who have seen it, but not easily conveyed in words.

"Almost all writers on the plague take notice of a peculiar cast of countenance, which to those who are conversant with the disease, is one of its best diagnostics. It was the state of the eyes, Dr. Russell remarks, which contributed chiefly to occasion that confusion of countenance which he does not attempt to describe, but from which, after repeatedly observing it, he could with some certainty pronounce whether the disease was the plague or not.

"The danger is very generally proportioned to the degree of this symptom. When the eyes resume the natural appearance, particularly when this happens after sweats, the prognosis is favorable. But in the form of the plague we are considering, this hardly ever happens. In the comatose the muddiness of the eyes is most remarkable. Their fierceness is most striking in those who labour under delirium, particularly the furious delirium; and it sometimes happens, that the coma and delirium, with the peculiar casts of countenance which accompany them, alternate with each other. In the delirious, however, there is still an appearance of muddiness, and the eyes retain some lustre in the comatose. These appearances of the eyes are less remarkable in children than in adults. Such is the best account I have been able to collect from the observations of those who were conversant with the disease, of that peculiar appearance of the eyes, which all who have seen the plague agree, so remarkably characterises it.

"The changes which take place in the eye are not always confined to its appearance only; the retina is sometimes much affected. The patients patients complain of feeing fparks, flafhes of fire, and various colours paffing before the eyes; this is only a greater degree of the fymptoms termed mufcæ volitantes. Deceptions of fight, however, are not frequent fymptoms in the plague. Deceptions of hearing are ftill more rare. Deafnefs, as in other fevers, is generally a favourable fymptom, but it feldom attends the plague. With refpect to the other fenfes, nothing particular is to be obferved. The depravation of the tafte, which is in fome meafure characteriftic of the plague, I have already had occafion to notice.

" The anxiety in many cafes is extreme, the patient conftantly changing his pofture, and foon finding the prefent as uneafy as the laft, fo that he is fometimes perpetually in motion. When this fymptom is confiderable, it affords a very unfavorable prognofis. The appearance it affumes when at its height, which generally indicates the approach of death, is defcribed by authors, who have termed it a mortal inquietude, in very ftrong terms: The patient inceffantly twifts his body as if in agony, but is incapable of giving any account of his feelings, fo that it is difficult to determine whether it is occafioned by a great degree of anxiety or fevere pain.

" The temperature, in the progrefs of the difeafe, is various. While the chills continue to recur, its increafe is not confiderable, and in the cafes where it is moft confiderable, it feldom equals that which we often meet with in common fynocha.

" The ftate of the pulfe is alfo various. In moft cafes after the firft days of the difeafe, in many after the firft hours, and in fome from the commencement, it is feeble and frequent. Sometimes it is remarkably hard and fmall, but regular, at other times it is irregular or intermitting, and at length fluttering.

" During the exacerbations, it often becomes full, open, and ftrong, as Dr. Ruffell expreffes it; after which it again finks; but in a more advanced ftage, a different change is obferved during the exacerbations, the pulfe becoming fo feeble that it can with difficulty be felt.

" It has been obferved of the pulfe in the plague, that though to a flight touch it is ftrong and full, it is often eafily compreffed; a ftate of the pulfe not readily accounted for, which is mentioned however by more than one author from their own obfervation. The moft ftriking fact relating to the ftate of the pulfe in the plague is, that it has often been obferved nearly natural while the other fymptoms indicated much danger.

" As the difeafe advances, the increafe of debility is generally indicated by a confiderable affection of the fpeech; in fome cafes amounting only to a degree of confufion and faltering, or a change of tone; in others the voice is greatly impaired or wholly loft. The affection of the voice appears the earlier, the greater the debility. When this is exceffive from the beginning, when, as Chenot obferves, ' Ægri erecti ftare aut federe impotes, proprio pondere labebantur,' a confiderable affection of the fpeech is generally obferved obferved on the firft night, or the fecond at fartheft. When the debility is lefs confiderable, it is delayed to the third day or later.

"The ftate of the tongue, as in other fevers, is various. It often retains the natural appearance throughout the greater part of the difeafe. Sometimes it is moift, and covered with thick mucus, at other times dry. 'Sometimes,' Dr. Ruffell obferves, 'it became parched with a yellow ftreak on each fide, and reddifh in the 'middle, but it never was obferved to form fo thick a fur or become 'of fo dark a colour as in the advanced ftages of fome other fevers. 'The drynefs or moiftnefs of the tongue,' he adds, 'rarely correfponded with the febrile fymptoms, for the tongue was often 'moift where the external heat was intenfe, and the pulfe indicated 'high fever; and on the contrary, parched where the fever in ap'pearance was very inconfiderable.'

"Vomiting, though more frequently obferved in lefs violent forms of the plague, fometimes attends this variety. The pain at the heart, fo frequently complained of, Dr Ruffell thinks fituated about the orifice of the ftomach. As it often accompanies vomiting, he was at firft led to believe, that it arofe from bile or other irritating matter in the ftomach. He found, however, that it was not relieved by the difcharge. It feems more than probable, from the nature of the fymptoms, that this pain proceeds from an inflammatory affection of the ftomach. The matter evacuated by vomiting is generally bilious. It is fometimes of a dark colour and mixed with blood; and it is not uncommon in the plague for worms to be thrown out by vomiting. Whatever be the appearance of the matter rejected, when vomiting occurs at an early period and returns at intervals, the prognofis is bad.

"Naufea without vomiting is a frequent fymptom. It does not feem to proceed from irritating matter in the ftomach, as repeated vomiting is not found to relieve it. There are few means more effectual for allaying naufea and vomiting than thofe which promote the perfpiration. It has been obferved of the plague, that if the repeated reaching occafions a moifture on the fkin, the naufea abates.

"A diarrhœa is apt to fupervene, fometimes during the firft days, more frequently at a later period. This is invariably a dangerous fymptom. The matter paffed by ftool is fimilar to that rejected by vomiting, often bilious, frequently with an evident admixture of blood; fometimes blood only is paffed. Chenot often met with dyfenteric purging in the plague.

"As there are few fevers in which purging is fo unfavorable, fo there are none in which conftipation appears to be lefs injurious. 'A number of the fick,' Dr. Ruffell obferves, 'were difpofed to be 'coftive throughout the difeafe, and fome had no ftool for feveral 'days, the popular dread of provoking a diarrhœa proving a bar 'to laxatives and even to fimple clyfters, which are readily ad'mitted at other times. The confequences of this fluggifhnefs of 'the bowels were by no means what might have been expected, 'for on comparing a number of cafes in which the body had been

'all

'all along regular, with others in which there had been no ftool, 'the former did not appear to have been particularly exempt 'from thofe fymptoms which might plaufibly have been imputed 'to coftivenefs in others.'

"The urine is often obferved in no refpect different from that of a perfon in health; at other times it is found pale, high coloured, clear, turbid, without fediment, or with a great deal, and fometimes more or lefs tinged with blood; in fhort, it affumes in different patients, or fometimes in the fame at different times, all the various appearances obferved in other fevers.

"There is no excretion of fuch confequence in the plague as that by the fkin. When the fkin remains parched, or when only flight, clammy, partial fweats appear, the prognofis is bad; when on the other hand a thin, general, and copious fweat takes place, it often proves more or lefs critical.

"Dr. Ruffell obferves, that the breath and perfpiration were feldom or never fetid. Other writers, however, have obferved, that they are often fetid to a great degree.

"As in many, perhaps in all the other exanthemata, epileptic fits now and then occur They are a rare fymptom in the plague. When they appear, they generally precede an eruption.

"Slight convulfive motions of the limbs and fubfultus tendinum are frequent; with refpect to other convulfive motions, hiccup rarely, and fneezing almoft never, attends the plague.

"Hemorrhagies are a common fymptom, and unlefs very moderate, generally indicate much danger. They are frequent, as appears from what has been faid, from the ftomach, inteftines, and kidneys. They are more common however from the nofe, and in women from the uterus, than from other parts. Thefe, particularly the hemorrhagy from the nofe, when they occur early in the difeafe, and the patient is young and plethoric, fometimes bring relief. At a later period, hemorrhagies are always unfavourable, and when they become profufe, the patient feldom recovers. It has been remarked, as might have been inferred a priori, that the blood which flows in thefe hemorrhagies is thinner in proportion as the difeafe is further advanced.

"Such are the fymptoms of the worft form of the plague. The ftrength gradually finks, till the pulfe impreffes the finger with only a weak, undulating, or tremulous motion, with frequent intermiffions. The furface, particularly on the extremities, becomes cold and covered with clammy moifture, the pulfe cannot be felt, and the patient calmly expires, or, as frequently happens in all idiopathic fevers, is carried off by convulfions.

[ To be continued. ]

